# Immune-Mediated Toxic Epidermal Necrolysis

**DOI:** 10.7759/cureus.9587

**Published:** 2020-08-06

**Authors:** Dinesh Keerty, Viktoriya Koverzhenko, Dalila Belinc, Katie LaPorta, Elizabeth Haynes

**Affiliations:** 1 Internal and Hospital Medicine, Moffitt Cancer Center, Tampa, USA; 2 Internal Medicine, Moffitt Cancer Center, Tampa, USA

**Keywords:** drug-related side effects and adverse reactions, pembrolizumab, toxic epidermal necrolysis (ten), stevens-johnson

## Abstract

The treatment of melanoma has advanced over time with the latest modalities being immune checkpoint blockade by programmed death receptor 1 and cytotoxic T-lymphocyte-associated antigen 4 inhibitors. Programmed death receptor 1 inhibitors have been noted to cause multi-system adverse reactions. The dermatological adverse events can range from pruritus to severe toxic epidermal necrolysis. We report a fatal case of toxic epidermal necrolysis secondary to nivolumab therapy. Checkpoint inhibitors are becoming the standard treatment option in many malignancies. Their safety profile is still evolving as more cases are being reported. Many individuals who are immunocompromised or undergoing concomitant treatment with combination therapy could develop significant overlapping toxicities. Physicians must be vigilant for dermatological complications that lead to opportunistic infections and sepsis.

## Introduction

Melanoma is the fifth most prevalent cancer in the United States [1]. The treatment of melanoma has advanced over time with the latest modalities being immune checkpoint blockade by programmed death receptor 1 (PD-1) inhibitors and cytotoxic T-lymphocyte-associated antigen 4 (CTLA-4) inhibitors [2]. Before the utilization of checkpoint inhibitors, the median overall survival for patients with metastatic melanoma was less than 12 months [3,4]. However, there are many side effects of checkpoint inhibitors, such as pneumonitis, hypophysitis, hepatitis, and rheumatological flairs [5]. The dermatological adverse events can range from pruritus and morbilliform exanthems to Stevens-Johnson syndrome (SJS), or toxic epidermal necrolysis (TEN) [6]. We report a fatal case of toxic epidermal necrolysis secondary nivolumab therapy in the treatment of melanoma. 

## Case presentation

**Figure 1:**
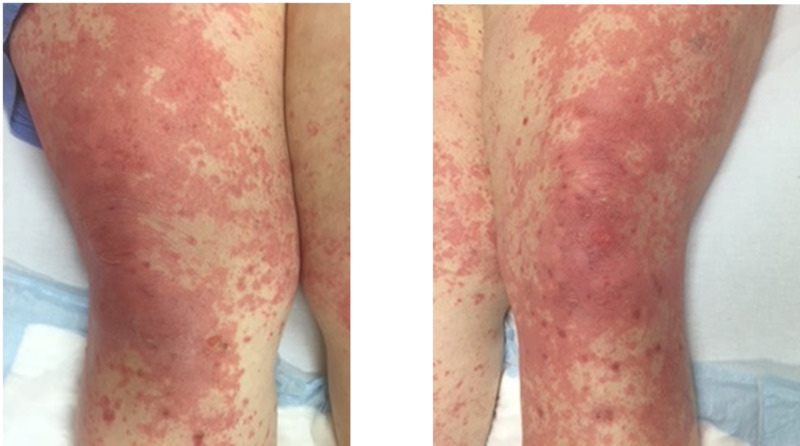
Bilateral thighs with erythematous macules Erythematous blanching macules coalescing into large patches diffusely

This is a case of a 50-year-old female with metastatic melanoma. She has a strong history significant for diabetes, hypertension, and morbid obesity with a body mass index of 50. She takes metformin for her diabetes. She was started on a combination therapy with ipilimumab and nivolumab. After her first dose, she developed a grade 2 maculopapular rash and was subsequently treated with a short steroid taper with methylprednisolone pack. Due to the rash, her treatment was changed to monotherapy with nivolumab. After two cycles of nivolumab, she developed an erythematous appearing lesion on her lower extremities. She presented to the clinic with worsening erythema. She was evaluated dermatology and had punch biopsies performed. She was started on prednisone 1 mg/kg (120 mg/day) for grade 3 immune-mediated drug eruption. She was also started on sulfamethoxazole-trimethoprim for pneumocystis pneumonia prophylaxis. She was discharged home for outpatient follow-up. Despite a higher dose of steroids, the rash continued to worsen over a week, becoming more confluent, painful, and intensely pruritic. There were no signs of ulceration, bullae, or pustules (Figure 1).  

**Figure 2:**
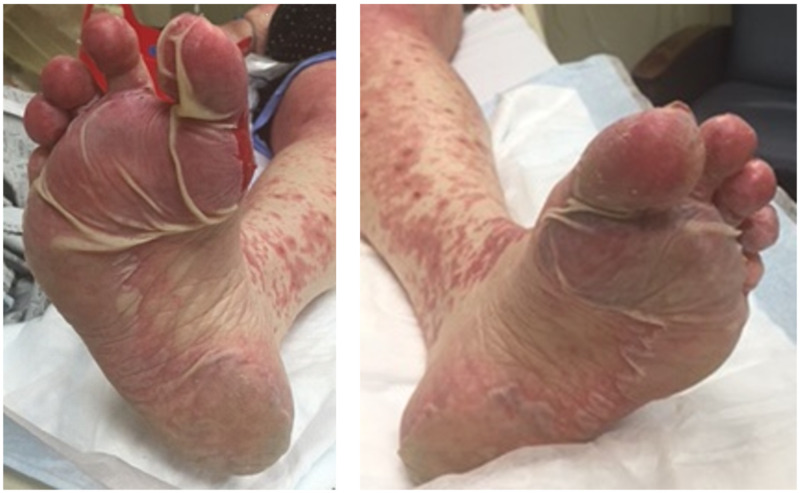
Desquamation of the soles Full thickness desquamation of plantar feet bilaterally

In the interim, the biopsy results showed interface dermatitis suspicious for drug eruption or erythema multiforme. She presented to the emergency department a week later with a severe, progressive skin rash that had now blistered all over. The blisters appeared on the soles of her feet and progressed towards her abdomen and upper extremities (Figure 2).

She also had blistering and sloughing of skin in her mouth and her labia. The patient had no sign of nasal, oropharyngeal, or vaginal bleeding. On examination, she had a positive Nikolsky sign and desquamation of buccal mucosa and plantar aspect of her feet. Due to the rapid blistering and sloughing of her skin, she was transferred to a tertiary hospital's burn unit. She developed concurrent bacterial sepsis from excessive desquamation and ultimately succumbed to her illness.

## Discussion

SJS or TEN is diagnosed based on the degree of skin involvement. For SJS, skin involvement is <10%, while it is often >30% for TEN [7]. Skin changes usually appear within the first week after exposure to the particular medication or could be delayed in some instances. It is followed by a period of flu-like prodrome, which can include fever, malaise, runny nose, or cough. The onset is abrupt, and it consists of tender/painful erythematous skin rash. The rash most commonly presents on the trunk with subsequent extension towards the face and limbs. These events usually occur within two to four days from the initial onset. The rash could be macular, erythematous, targetoid, or blistering in appearance. The blisters coalesce to form sheets of skin desquamation, exposing the underlying dermis. The histopathology typically shows keratinocyte necrosis with minimal inflammation. A direct immunofluorescence test on the skin biopsy is usually negative, as was similarly noted in our patient. This indicates that the disease is not due to the deposition of antibodies in the dermal layers [7]. 

Toxic epidermal necrolysis is a life-threatening epidermal desquamation of various mucosal surfaces due to CD8^+^ T lymphocyte-induced apoptosis of epithelial keratinocytes. It can rapidly involve gastrointestinal, respiratory, and genitourinary tracts [8]. A low-grade rash is the most common dermatological adverse event that was reported from the use of PD-1 inhibitors and CTLA-4 inhibitors [9]. The half-life of nivolumab, 17 to 21 days, could lead to prolonged toxicity. Most grade 3 to 4 skin toxicities have been reported during the post-trial evaluation, usually due to higher doses of immunotherapy [10]. Another randomized phase III study reported the occurrence of grade 3 to 4 rash in only two cases of advanced-stage melanoma who were treated with combination nivolumab and ipilimumab [11]. Goldinger et al. demonstrated that anti-PD-1 antibodies frequently cause adverse cutaneous reactions. They noticed gene expression profiling depicted cases of an TEN-like pattern, which suggests that PD-1/PD-L1 (programmed cell death ligand 1) interaction is required to preserve epidermal integrity [12]. There is growing evidence of the ability of anti‐PD‐1 antibodies to induce TEN without the classic clinical morphology [13]. 

The management of TEN/SJS involves permanent discontinuation of immunotherapy, an urgent dermatology consultation, skin biopsy, and initiation of steroids. Initially, prednisone or methylprednisolone 1-2 mg/kg/day is utilized with the patient being monitored in the hospital. After three days, if there is no significant improvement, rituximab or intravenous immunoglobulins (IVIG) can be added [14]. Etanercept, a tumor necrosis factor alpha (TNF-alpha) agonist, has also been used in the treatment of TEN with a median healing time of eight to nine days but requires immediate initiation [15]. 

In a study by Simonaggio et al., patients who experienced a grade 2 or higher immune-related adverse event with an anti-PD-1 and subsequently were re-challenged with anti-PD-1, 55% experienced a second adverse event. Anti-PD-1 was deemed acceptable since the risk vs. benefit of clinical remission was improved on re-challenge with anti-PD-1 treatment [16]. Criteria for outpatient treatment of adverse events are based on patient tolerance and grade of the adverse event.

When reviewing her case, we evaluated her SCORe for Toxic Epidermal Necrosis (SCORTEN) and also if her anti-diabetic medications could have played a role in her treatment efficacy. Her SCORTEN score was 5 indicating greater than 90% mortality. SCORTEN has been deemed an accurate scoring system for estimation of mortality among TEN patients treated in burn centers [17]. Metformin has been noted to downregulate PD-1 expression in tumor cells and showed increased efficacy of immunotherapy [18]. In our patient during re-challenge, she developed a recurrence of rash possibly due to weight-based dosing of immunotherapy. At the tertiary hospital, she continued with IV steroids, IVIG, and infliximab, a TNF-alpha agonist. She succumbed to superimposed bacterial sepsis.

## Conclusions

Checkpoint inhibitors are being utilized in the treatment of many malignancies, both on- and off-label. Their adverse reactions are still being learned as more cases get reported. Patients on concomitant treatment with combination therapy could develop significant overlapping toxicities. Physicians must maintain a high degree of clinical suspicion to establish a diagnosis, and to obtain a biopsy early during treatment with anti-PD-1 therapies. Since the treatment relies on the use of high-dose steroids, any delay in diagnosis and treatment could lead to the development of opportunistic infections and/or sepsis that can cause an untimely demise. 

## References

[1] (2020). Melanoma of the skin - cancer stat facts. https://seer.cancer.gov/statfacts/html/melan.html.

[2] Ugurel S, Kiecker F, Fröhling S (2017). Fulminant response to combined checkpoint inhibition with ipilimumab plus nivolumab after failure of nivolumab monotherapy in metastatic melanoma. Eur J Cancer.

[3] Rothermel LD, Sarnaik AA, Khushalani NI, Sondak VK (2019). Current immunotherapy practices in melanoma. Surg Oncol Clin N Am.

[4] Lipson EJ, Drake CG (2011). Ipilimumab: an anti-CTLA-4 antibody for metastatic melanoma. Clin Cancer Res.

[5] Trinh S, Le A, Gowani S, La-Beck NM (2019). Management of immune-related adverse events associated with immune checkpoint inhibitor therapy: a minireview of current clinical guidelines. Asia Pac J Oncol Nurs.

[6] Tattersall IW, Leventhal JS (2020). Cutaneous toxicities of immune checkpoint inhibitors: the role of the dermatologist. Yale J Biol Med.

[7] (2020). Stevens-Johnson syndrome/toxic epidermal necrolysis. https://dermnetnz.org/topics/stevens-johnson-syndrome-toxic-epidermal-necrolysis/.

[8] Griffin LL, Cove-Smith L, Alachkar H, Radford JA, Brooke R, Linton KM (2018). Toxic epidermal necrolysis (TEN) associated with the use of nivolumab (PD-1 inhibitor) for lymphoma. JAAD Case Rep.

[9] Ansell SM, Lesokhin AM, Borrello I (2015). PD-1 blockade with nivolumab in relapsed or refractory Hodgkin's lymphoma. N Engl J Med.

[10] Brahmer JR, Tykodi SS, Chow LQ (2012). Safety and activity of anti-PD-L1 antibody in patients with advanced cancer. N Engl J Med.

[11] Larkin J, Chiarion-Sileni V, Gonzalez R (2015). Combined nivolumab and ipilimumab or monotherapy in untreated melanoma. N Engl J Med.

[12] Goldinger SM, Stieger P, Meier B, Micaletto S, Contassot E, French LE, Dummer R (2016). Cytotoxic cutaneous adverse drug reactions during anti-PD-1 therapy. Clin Cancer Res.

[13] Vivar KL, Deschaine M, Messina J (2017). Epidermal programmed cell death-ligand 1 expression in TEN associated with nivolumab therapy. J Cutan Pathol.

[14] Thompson J, Schneider B, Brahmer J (2019). Management of immunotherapy-related toxicities, Version 1.2019. J Natl Compr Canc Netw.

[15] Paradisi A, Abeni D, Bergamo F, Ricci F, Didona D, Didona B (2014). Etanercept therapy for toxic epidermal necrolysis. J Am Acad Dermatol.

[16] Simonaggio A, Michot JM, Voisin AL (2019). Evaluation of readministration of immune checkpoint inhibitors after immune-related adverse events in patients with cancer. JAMA Oncol.

[17] Cartotto R, Mayich M, Nickerson D, Gomez M (2008). SCORTEN accurately predicts mortality among toxic epidermal necrolysis patients treated in a burn center. J Burn Care Res.

[18] Verdura S, Cuyàs E, Martin-Castillo B, Menendez JA (2019). Metformin as an archetype immuno-metabolic adjuvant for cancer immunotherapy. Oncoimmunology.

